# Independent Whole-Genome Duplications Define the Architecture of the Genomes of the Devastating West African Cacao Black Pod Pathogen *Phytophthora megakarya* and Its Close Relative *Phytophthora palmivora*

**DOI:** 10.1534/g3.120.401014

**Published:** 2020-04-30

**Authors:** Abraham Morales-Cruz, Shahin S. Ali, Andrea Minio, Rosa Figueroa-Balderas, Jadran F. García, Takao Kasuga, Alina S. Puig, Jean-Philippe Marelli, Bryan A. Bailey, Dario Cantu

**Affiliations:** * Department of Viticulture and Enology, University of California Davis, California,; † Sustainable Perennial Crops Laboratory, USDA/ARS, Beltsville, Maryland,; ‡ Crops Pathology and Genetics Research Unit, United States Department of Agriculture–Agricultural Research Service, Davis, California,; § Subtropical Horticultural Research Station, USDA/ARS, Miami, FL 33158, and; ** Mars Wrigley Plant Sciences Laboratory, 95616 Davis California

**Keywords:** plant diseases, effectors, RxLR motif, transposable elements, whole-genome duplication, oomycetes

## Abstract

*Phytophthora megakarya* and *P. palmivora* are oomycete pathogens that cause black pod rot of cacao (*Theobroma cacao*), the most economically important disease on cacao globally. While *P. palmivora* is a cosmopolitan pathogen, *P. megakarya*, which is more aggressive on cacao than *P. palmivora*, has been reported only in West and Central Africa where it has been spreading and devastating cacao farms since the 1950s. In this study, we reconstructed the complete diploid genomes of multiple isolates of both species using single-molecule real-time sequencing. Thirty-one additional genotypes were sequenced to analyze inter- and intra-species genomic diversity. The *P. megakarya* genome is exceptionally large (222 Mbp) and nearly twice the size of *P. palmivora* (135 Mbp) and most known *Phytophthora* species (∼100 Mbp on average). Previous reports pointed toward a whole-genome duplication (WGD) in *P. palmivora*. In this study, we demonstrate that both species underwent independent and relatively recent WGD events. In *P. megakarya* we identified a unique combination of WGD and large-scale transposable element driven genome expansion, which places this genome in the upper range of *Phytophthora* genome sizes, as well as effector pools with 1,382 predicted RxLR effectors. Finally, this study provides evidence of adaptive evolution of effectors like RxLRs and Crinklers, and discusses the implications of effector expansion and diversification.

Cacao (*Theobroma cacao* L.) beans are the backbone of the global chocolate industry, which is valued at over 100 billion US Dollars annually (“Zion Market Research” 2018). Cacao is also the major cash crop for millions of small holder farmers in the tropics with a total harvest of 5.2 million tons (FAO, 2017). However, global cacao production is threatened by multiple diseases that negatively impact yield and quality (Marelli *et al.* 2019). Black pod rot is responsible for more than half the total reported crop loss, destroying the equivalent of 0.87 million metric tons or 2 billion US dollars’ worth of dried cacao beans annually (Figure 1A) (Marelli *et al.* 2019). Black pod rot is caused by multiple *Phytophthora* species, among which *P. palmivora* is the most widespread and *P. megakarya*, currently confined to West and Central Africa, is the most destructive, causing up to 90% crop loss if not controlled (Figure 1B)(Ali *et al.* 2016). In recent years, *P. megakarya* has largely displaced *P. palmivora* as a major cause of black pod rot in some African nations (Ali *et al.* 2017).

**Figure 1:**
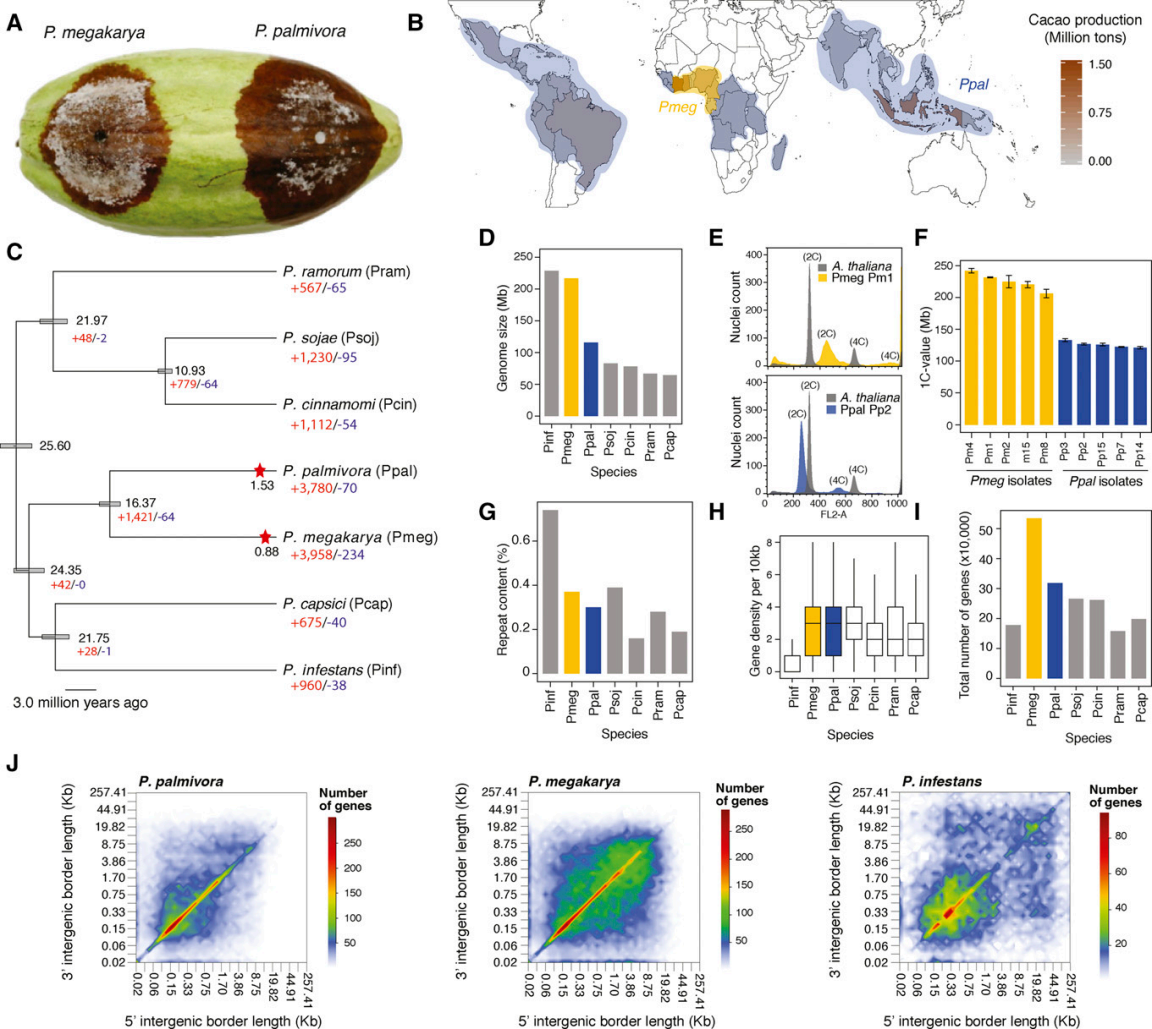
(A) Black pod rot symptoms by *P. megakarya* and *P. palmivora*. (B) Approximate geographical distribution of *P. megakarya* (yellow area) and *P. palmivora* (blue area). (C) Clock-calibrated phylogenetic tree with estimated times of divergence and WGD duplication events in *P. palmivora* and *P. megakarya* in million years ago, with numbers of families expanded and contracted across multiple *Phytophtora* spp. (D) Genome size of multiple *Phytophthora* spp. (E) Example run of the flow cytometry using *Arabidopsis* as control. (F) Average estimated 1C-values from flow cytometry. (G) Repeat content of multiple *Phytophthora* spp. (H) Boxplot showing the distribution of gene density per 10 Kb. (I) Total number of genes per species. (J) Intergenic space heatmap of *P. palmivora*, *P. megakarya* and *P. infestans*. *Pinf*: *P. infestans*, *Pmeg*: *P. megakarya*, *Ppal*: *P. palmivora*, *Psoj*: *P. sojae*, *Pcin*: *P. cinnamomi*, *Pram*: *P. ramorum* and *Pcap*: *P. capsici*.

The genus *Phytophthora* includes more than 120 known species of filamentous oomycetes (Kroon *et al.* 2012) and an estimated 300-500 species not yet discovered (Brasier 2007). Most of the known species are pathogenic to plants and some have devastating effects on crops and natural forests. There is expanding interest in *Phytophthora* species due to their economic and environmental impact. This has driven the release of genome assemblies for at least 26 *Phytophthora* species in the last 10 years. These genome assemblies have helped identify gene families critical to infection processes. These gene families include effectors like crinklers (CRNs), necrosis inducing proteins (NPPs), and proteins characterized by an N-terminal Arg- Xaa-Leu-Arg motif (RxLRs), all associated to pathogenicity and host specificity (Morgan and Kamoun 2007; Jiang *et al.* 2008; Haas *et al.* 2009). Studies of *Phytophthora* genomes have shown that they contain significant amounts of repetitive elements. Transposable elements (TEs), specifically, play a significant role in the evolution of *Phytophthora* (Judelson 2012; Kasuga *et al.* 2016; Ali *et al.* 2017; Elliott *et al.* 2018). For example, in *P. ramorum* transposons have been associated with the generation of genotypic diversity by introducing chromosomal breakpoints (Dale *et al.* 2019).

Although *P. megakarya* and *P. palmivora* are closely related and belong to clade 4 in the *Phytophthora* phylogeny (Figure 1C) (Blair *et al.* 2008), they possess different numbers and sizes of chromosomes (9-12 smaller chromosomes in *P. palmivora*, 5-6 large chromosomes in *P. megakarya*) and have different geographic distributions, host ranges, and aggressiveness (Sansome *et al.* 1975; Brasier and Griffin 1979). Southeast Asia (Mchau and Coffey 1994) and Central Africa (Nyassé *et al.* 1999) are the centers of origin of *P. palmivora* and *P. megakarya*, respectively. Thus, they provide an opportunity to study the post-speciation divergent evolutionary trajectories of two pathogens that parasitize the same host with different levels of virulence. The interactions between each pathogen and cacao are likely to have been relatively recent. Initial draft genome assemblies for both species based on short-read sequencing suggested a whole-genome duplication (WGD) in *P. palmivora* and a retroelement-based expansion in the genome of *P. megakarya*, resulting in particularly high numbers of RxLRs (Ali *et al.* 2017). However, complete genome assemblies for the two pathogens will be crucial to understanding their evolution and diversity and dissecting the mechanisms and adaptations that drive pathogenicity of black pod rot in cacao.

In this study, we assembled the diploid genomes of multiple isolates of *P. megakarya* and *P. palmivora* using long reads. We found that the genome assembly size of *P. megakarya* was nearly twofold larger than previously reported (Ali *et al.* 2017), and validated the unexpected genome size by flow cytometry across multiple isolates. Further, we found strong evidence of WGDs in both species, as well as dramatic gene family expansions in *P. megakarya* that likely explain the difference in the genomes of two species. We then examined highly expanded gene families in *P. megakarya* and *P. palmivora* compared to other *Phytophthora* species, finding associations with transposable elements and large number of effectors. Finally, we re-sequenced 28 additional genotypes from cacao-producing countries and report evidence of adaptive evolution in well-known effectors that have recently increased in number by WGD and gene family expansion.

## Materials And Methods

### Biological material

*P. megakarya* and *P. palmivora* isolates were obtained from collections held by *USDA*, *Beltsville*, *USA* and Sydney Institute of Agriculture, Australia. Isolates were initially collected from black pod infected cacao in Western Africa, Central America, Southeast Asia and Papua New Guinea and identified to species as previously described (Ali *et al.* 2016). Isolates of *Phytophthora* spp. and their sources are listed in Table S1.

### Isolation of P. megakarya and P. palmivora gDNA for short-read sequencing

For DNA extraction, *P. megakarya* and *P. palmivora* isolates were grown for seven days on 20% clarified V8 agar (CV8). 2-3 agar plugs (0.25 cm^2^) from the cultures were then transferred to 50 ml falcon tubes containing 20 ml liquid CV8 and grown at room temperature while shaking at 100 rpm. Cultures were incubated for 5-10 days. The mycelia were washed with sterile water and collected by centrifuging at 20,000 g for 10 min, followed by flash freezing in liquid nitrogen and freeze dried. Approximately 0.1 g of freeze-dried mycelia were pulverized in a mortar and pestle under liquid nitrogen. DNA was extracted by adding the ground mycelia to a tube containing 10 ml of pre-warmed (65°) modified CTAB buffer (3% cetyltrimethylammonium bromide, 100 mM Tris, pH 8, 20 mM EDTA, pH 8, 1.4 M NaCl, 1% PVP 40,000, 0.2% 2-mercaptoethanol, 80 µg ml^-1^ proteinase K) followed by incubation at 65° for 1 h. Each sample was extracted twice with 10 ml chloroform and the upper phase was transferred to a fresh tube. DNA was precipitated by adding 5 ml 7.5M ammonium acetate and 20 ml absolute ethanol to each tube and holding on ice for 60 min followed by centrifuging at 18,000 g for 15 min. The DNA pellet was washed with 70% ethanol, air-dried and re-suspended in 500 µl EB buffer (QIAGEN, USA). For RNase treatment, samples were treated with 0.5 µg/µl RNase A (Invitrogen, USA) enzyme followed by incubation at 37° for 15 min. Samples were again subjected to chloroform extraction and ethanol precipitation as mentioned above and DNA was re-suspended in 100 µl EB buffer.

### Isolation of P. megakarya and P. palmivora gDNA for SMRT sequencing

High molecular weight gDNA was extracted using a modified protocol described by Stoffel *et al.* (Stoffel *et al.* 2012). Freeze dried mycelia (≃ 0.25 g) were ground in a mortar and pestle under liquid nitrogen and transferred to 25 ml chilled stainless-steel jar. The jar was submerged in liquid nitrogen for few minutes and transfer to TissueLyser II (Qiagen, USA). Samples were further ground for 45 s at 30 Hz. Grind tissue were transfer to 50 ml falcon tube containing 15 ml of pre-warmed 2X extraction buffer (100 mM Tris–HCl pH 8.0, 1.4 M NaCl, 20 mM EDTA, 2% w/v CTAB, 10 μl/ml β-mercaptoethanol), gently mixed and incubated at 65° for 1 h. The tube was centrifuged at 10,000 rpm for 10 min to remove the cellular debris and the supernatant was transferred to a new tube containing 15 ml chloroform:isoamyl alcohol (24:1) (ChlA), gently mixed, and centrifuged at 10,000 rpm for 30 min. The aqueous phase was then transferred to a new tube containing 7.5 ml of 5 M NaCl and equal volume of ChlA was added and mixed gently and centrifuged at 10,000 rpm for 10 min. The aqueous phase was then transferred to 3 Oak Ridge tubes and 4 to 5 volumes of precipitation buffer (50 mM Tris–HCl pH 8.0, 10 mM EDTA, 1% w/v CTAB) were added. The sample was incubated overnight at room temperature to precipitate the DNA and then centrifuged at 14,000 rpm for 30 min. The DNA pellet was washed with 5 ml dH_2_O and pulled in to one Oak Ridge tube and centrifuged at 14,000 rpm for 10 min. DNA pellet was dissolved in 500 µl of 1.5 M NaCl and 1 µg/µl RNaseA (Invitrogen, USA) was added to the pellet and incubated at 37° for 45 min. A chloroform extraction was performed as above to remove RNaseA and any additional contaminants. The aqueous phase was collected, and DNA was precipitated with 3 volumes of absolute ethanol, followed by centrifugation for 30 min at 14,000 rpm and washed with 70% ethanol. The air-dried pellet was re-suspended in 100 μl EB buffer (QIAGEN, USA). The sample was diluted 1:25 and concentration of the gDNA was quantified with a Qubit 3.0 fluorometer using a Qubit dsDNA HS Assay Kit (Thermo Fisher Scientific, USA). The quality of the extracted gDNA was assessed using a NanoDrop UV/Vis spectrophotometer and 1% (w/v) agarose gel. Approximately 1 µg of the gDNA was run on a 0.75% pippin pulse (Sage Science, USA) gel to examine the integrity and molecular weight of the gDNA.

### Isolation of RNA From mycelia and zoospores

For RNA extraction from mycelia (*P. megakarya* and *P. palmivora* isolates Pm1 and Pp2 respectively), 2-3 agar plugs from a V8 agar plate culture were transferred to 250 ml conical flasks containing 50 ml liquid CV8. Liquid cultures were grown 7 days at room temperature (≃25°) with shaking at 100 rpm. Mycelia were washed with sterile water and collected by centrifuging at 20,000 g for 10 min followed by flash freezing in liquid nitrogen and freeze drying. Freeze-dried mycelia were ground in a mortar and pestle in liquid nitrogen and transferred to a 50 mL centrifuge tube containing 15 mL of 65° extraction buffer (Bailey *et al.* 2005). The remaining extraction procedure was conducted as described (Bailey *et al.* 2013). Using a NanoDrop spectrophotometer (Thermo Scientific, USA), RNA concentrations were determined based on absorbance at 260 nm and purity was estimated by the 260/280 and the 260/230 ratios.

For RNA extraction from zoospores, cultures of Pm1 and Pp2 were grown on a CV8 agar plate for 7 days under constant darkness at 25° and then transferred to constant light (200 lux) for 4-5 days. For zoospore release, each plate was flooded with cold sterile water (4°) and kept at 4° for 45 min then transferred to 28° for 28 min. The zoospore suspension was transferred to sterile 50 ml falcon tubes and collected by centrifuging at 15,000 g for 5 min. Zoospore pellets were transferred to a mortar and pestle and ground in liquid nitrogen. RNA extraction was carried out as described for mycelia.

### RNA extraction from infected plant material

Harvested pods of the susceptible cacao clone ‘Catongo’ were cut into 2.5 X 2.5 cm pieces; the inner core materials were removed and the pieces were surface-sterilized with 6% (vv^-1^) bleach (Clorox, USA) for 90 s followed by three rinses with sterile distilled water. Pieces were placed in sterile plastic containers (20 X 10 X 6 cm) lined with sterile tissue paper soaked in 0.7 mM benzimidazole solution (as a senescence retardant) at the bottom. For inoculation, 1 cm^2^ sterile Whatman no. 2 filter papers were soaked in the zoospore solutions (10^5^ zoospores ml^-1^) and placed in the middle of the exterior part of each husk piece. Control husk pieces were treated with filter paper soaked in sterile water. Containers were covered and incubated at 25° with 50% relative humidity under 12 h light (200 lx) and dark cycles. At 15 and 36 h post inoculation, the Whatman filter papers were removed and the husk pieces flash frozen in liquid nitrogen followed by freeze drying. For RNA isolation, freeze dried material was ground finely and approximately 0.05 g material transferred to a 50 mL centrifuge tube containing 15 mL of 65° extraction buffer and RNA extraction was carried out as described for mycelia.

### Whole genome and transcriptome libraries preparation

For preparation of DNAseq libraries, Bead-cleaned genomic DNA was randomly sheared to around 450 bp with a Covaris E220 sonicator. Sheared DNA was end-repaired, A-tailed and ligated to single adapters using the Kapa LTP library prep kit (Kapa Biosystems). RNAseq libraries were prepared using the Illumina TruSeq RNA sample preparation kit v.2 (Illumina, CA, USA), following Illumina’s protocol (Low-throughput protocol) and barcoded individually. Final libraries were evaluated for quantity and quality with the High Sensitivity chip in a Bioanalyzer 2100 (Agilent Technologies, CA) and Qubit (Invitrogen, CA). DNA libraries were submitted for sequencing at 150-bp paired-end mode on an Illumina HiSeq4000 sequencer (Novogene Co. Ltd., Beijing, China).

### SMRTbell libraries preparation

When needed, 200 µl of HMW gDNA at a concentration of 100 ng/µl were fragmented using a 26G blunt needle (SAI Infusion Technologies). gDNA shearing was done by aspirating the entire volume and passing the sample through the 26G blunt needle fifteen times. After shearing, sample was cleaned and concentrated using 0.45X AMPure PB beads and size distribution of the sheared gDNA fragments was evaluated using pulse field gel electrophoresis (Pippin pulse, Sage Science) prior to libraries preparation. SMRTbell template libraries from Pm1 and Pp2 isolates were prepared with 6-12 µg of sheared DNA using SMRTbell Template Prep Kit (Pacific Biosciences) following the manufacturer’s instructions. 30 μl of SMRTbell template was loaded into the Sage Blue Pippin for size selection and a cutoff range of 17-50 kbp was selected. Size selected library was cleaned with 1X AMPure PB beads and a repair DNA damage treatment was performed. After DNA repair, a new clean up step with 1X AMPure PB beads was done. A total of 6 SMRT cells per isolate were sequenced on the PacBio sequel system (Novogene Co. Ltd., Beijing, China). SMRTbell template libraries from Pm4, Pm15, Pp3, and Pp15 isolates were prepared with 6 µg of sheared DNA using the SMRTbell Express Template Prep Kit v2.0 (Pacific Biosciences) following the manufacturer’s instructions. A cutoff range of 17-50 kbp was chosen for size selection. Size selected library was cleaned with 1X AMPure PB beads. 4 SMRT cells per isolate were sequenced on the PacBio sequel system using a V3 chemistry (Genome center, UCDavis).

### Genome assembly

Assembly of the genomes was performed using SMRT reads with FALCON-Unzip ver. 2017.06.28-18.01 (Chin *et al.* 2016) adopting the custom pipeline published in (Minio *et al.* 2019). The pipeline code is available at https://github.com/andreaminio/FalconUnzip-DClab. Before performing error correction of the raw reads, repetitive regions were marked using the TANmask and REPmask modules from the DAmasker v1.0 (Myers 2014), reducing the complexity of the read-to-read alignment phase. After error-correction, reads were again marked before proceeding with the assembling phase. This additional repeat masking step increased assembly contiguity by reducing the complexity of the overlap graph. FALCON was performed using different thresholds on seed-reads minimum length for overlap stage (length_cutoff_pr parameter) in order to find the least fragmented primary assembly. Parameter set include: “falcon_sense_skip_contained = TRUE”, “falcon_sense_option =–output_multi–min_idt 0.70–min_cov 4–max_n_read 400”, “length_cutoff_pr = 27000”, “ovlp_DBsplit_option = -x500”, “ovlp_HPCdaligner_option = -mtan -mrep2 -v -B128 -M60 -t60 -k20 -h256 -e.9 -l1000 -s100 -T16”, and “overlap_filtering_setting =–max_diff 100–max_cov 400–min_cov 3”. Unzip procedure for haplotype phasing was carried out with default parameters (Chin *et al.* 2016), followed by polishing of primary contigs and haplotigs with Arrow (from ConsensusCore2 v.3.0.0) using long reads. Primary contigs were scaffolded using SSPACE-Longreads v.1.1 (Boetzer and Pirovano 2014), followed by gap closing with PBJelly (PBsuite v15.8.4; (English *et al.* 2012, 2014)). To assess the genome assembly length, SMRT reads of *P. megakarya* isolate Pm1 and *P. palmivora* isolate Pp2 were also assembled using two other alternative assemblers, Canu v1.8-14 (Koren *et al.* 2017)) and WTDBG2 v2.3 (Ruan and Li 2020). Canu was performed separately by setting an expected genome size of 215 Mbp and 115 Mbp, error correction, trimming and assembly parameters are: “Error correction: minReadLength=1000 minOverlapLength=500 corOutCoverage=80”, “Trimming: minReadLength=1000 minOverlapLength=500”, and “Assembly: correctedErrorRate=0.04 minReadLength=1000 minOverlapLength=500”. WTDBG2 was performed with the parameters include “-S 4 -p 21 -k 0 -e 4 -L 5000” and consensus called with wtpoa-cns algorithm. Assembled sequences were then polished and scaffolded using long reads with Arrow (from ConsensusCore2 v.3.0.0) and SSPACE-Longreads v.1.1 respectively. The gene space completeness of the genome assemblies was evaluated using the BUSCO genes (Simão *et al.* 2015), specifically using BUSCO v1.22 and the “eukaryota_odb9” database.

### Same scaffold gene block identification and validation

Fusion events in between colinear gene blocks were selected to design primers. A maximum length of 1000 bp was used as a threshold to select the candidates for the primer design. Those sequences were extracted from the primary alignment of both species using 2000 bp upstream and downstream the fusion event. This sequences individually served as input for Primer3plus online software (Untergasser *et al.* 2007) in detection mode. For each Fusion event a total of 5 primers pairs were generated and tested *in-silico* with the program Degenerate In-Silico PCR (dispr, https://github.com/douglasgscofield/dispr). All the primers were tested against the primary assembly of the corresponding specie allowing 1 mismatch in the first 3 bases in the 5′ of the primers, as well as product size range in between 200 bp and 3000 bp. The final primers sets were selected based on a specificity of the pair and the length of the product (maximum 1300 bp). The selected primers were used in a PCR with the corresponding species DNA. The reaction mix was prepared with 1X OneTaq Standard Reaction Buffer (New England Biolabs, Ipswich, Massachusetts), 1 mg/mL BSA, 0.2 mM dNTPs, 0.2 μM of each primer, 1.25 units of OneTaq DNA Polymerase (New England Biolabs, Ipswich, Massachusetts) and 1ng of DNA in a 25μL reaction, Nuclease free water was used as negative control of the reaction. The thermocycling profile (Veriti thermal cycler, Applied Biosystems) was set to an initial denaturation at 94° for 4 min, 31 cycles at 94° for 30 s, 62° for 30 s, and 68° for 1.16 min., and a final extension at 68° for 10 min. The amplicons were checked in an 1.5% (w/v) agarose with Apex Safe DNA Gel Stain (Apex Bioresearch Products), using 100 bp DNA ladder (New England Biolabs, Ipswich, Massachusetts). The running conditions were 80 V for 50min.

### Nuclei size content inference by flow cytometry

The details of the procedure can be found elsewhere (Malar *et al.* 2019). In brief, stationary grown mycelia in test tubes containing 5 mL filter-sterilized 5% clarified V8 broth for 7 days at 25° were harvested. Approximately 1 mg of dry blotted sample and five flower buds of *Arabidopsis thaliana* Col-0 were then combined and co-chopped in a Petri dish containing 500 μL extraction buffer (Cystain PI absolute P Kit, Sysmex America Inc.), and the suspension was filtered through a 10 μm filter (CellTrics, Sysmex America Inc.) and 2 mL of Propidium Iodine staining solution was added (Doležel and Bartoš 2005; Bertier *et al.* 2013). Measurements were done on a Becton Dickinson FACScan (Franklin Lakes, NewJersey) equipped with a 488 nm laser and a 585/42 nm band pass filter. Three biological replicates were measured per isolate. The data were analyzed using FlowJo v.10 (https://www.flowjo.com/solutions/flowjo) and DNA content was inferred using a linear regression with the ratios between the peak positions of *Phytophthora* sample and the *Arabidopsis* size standard (1C = 157 MB) (Doležel and Bartoš 2005).

### Gene prediction

RNAseq paired-end reads of 150 bp in length and were trimmed with Trimmomatic v0.36 (Bolger *et al.* 2014) with a 4-base wide sliding window, cutting when the average quality per base drops below 15 and a minimum length of 100 bp. Four biological replicates of each condition (*i.e.*, mycelium, zoospores, 15 hpi or 36 hpi) were merged and assembled separately by Trinity v2.6.5 (Grabherr *et al.* 2011) with the “–normalize_reads” option to create four transcriptomes per species. The assembled transcripts were then stringently mapped with PASA v2.0.2; (Haas *et al.* 2003) with a minimum percentage aligned of 95% and a minimum average percentage identity of 90% using the GMAP v2015-11-20 (Wu and Watanabe 2005) and BLAT v36x2 (Kent 2002) aligners to each genome. Candidate coding regions were then searched by TransDecoder v3.0.1 (Haas *et al.* 2013) in the assembled and aligned transcripts within the PASA pipeline. The alignments of best scoring transcripts were used to create a high-quality set of transcripts. The splicing information of this high-quality transcripts were used as hint for the gene predictor BRAKER1 v1.9 (Hoff *et al.* 2016) with the “–fungus” option to allow for potential overlapping genes on the softmasked genome assemblies. The resulting protein-coding genes were then taken through the PASA gene refinement pipeline, in which the initial Trinity assembled reads were mapped with more relaxed parameters (minimum alignment of 90% and minimum average identity of 85%) to polish the gene predicted models. To filter potential TEs from the predicted gene a series of filters were imposed. First, any gene that overlapped 90% or more with transposable element predicted by RepeatMasker was removed. Then, all the genes with a hit to an HMM repeat model of Dfam v3.0 (Hubley *et al.* 2016) was also removed. Also, genes with descriptions from BLAST2GO (see below) matching “transposon”, “transposable”, “transposase”, “retrov” or “helitron”; or with PFAM domains matching Pfam “Integrase” “RVT_”, “rve”, “Retrotrans”, “gag”, ”Chromo”, “RT_”, “Helitron” or “DDE_” were filtered out. Finally, a manual filtering of genes based other functional annotations of repeats was done. Gene families were created with a reciprocal BLASTP with an e-value of 1e-10 and clustered with MCL v14-137 (Enright *et al.* 2002) with and inflation value of 1.5. Protein coding predicted genes can be found in File S1.

### Gene functional annotation

The filtered proteins were aligned with BLASTP v2.6.0+ (Camacho *et al.* 2009) to the whole “RefSeq” protein database. The resulting alignments were used as input into Blast2GO v4.1.7 (Götz *et al.* 2008) and the proteins descriptions were extracted. The predicted proteins were also searched the Pfam database v32.0 (El-Gebali *et al.* 2019) using and a domain e-value cutoff of 0.001. Carbohydrate-active enzymes (CAZymes) were annotated using dbCAN2 (Zhang *et al.* 2018) with default parameters, and using only annotations validated by two of three tools. Candidate biosynthetic gene clusters were predicted with AntiSMASH Fungal v4.2.0 (Blin *et al.* 2017). Proteins were classified as secreted if they had a signal secretion peptide predicted by SignalP v4.1 (Petersen *et al.* 2011), but did not have a transmembrane domain (TM) in the first 60 amino acids or more than 2 TMs in total predicted by TMHMM v2.0 (Krogh *et al.* 2001), and if the protein did not have a mitochondrial targeting peptide (mTP) predicted by TargetP v1.1 (Emanuelsson *et al.* 2000). All functional annotations can be found in File S1.

### Repeat prediction

An initial run of RepeatMasker v4.0.6 (Smit *et al.* 2013–2015) with the standard library was done in the new *P. megakarya* and *P. palmivora* assemblies. The repeats predicted by this run were combined with the repeats extracted from multiple published *Phytophthora* masked assemblies (Table S2) to create a new database using the *de-novo* repeat predictor RepetModeler v1.0.11 (Smit and Hubley 2008–2015). The classified consensus sequences were then used as a custom library for another run of RepeatMasker in the *P. megakarya* and *P. palmivora* assemblies to predict the final repeats (File S2).

### Prediction of effectors

RxLR protein effectors from previously published *Phytophthora* (Table S2) species were classified in tribes using a reciprocal BLASTP v2.6.0+ with a cutoff e-value of 0.01, followed by a clustering with MCL using an inflation value of 1.5. The genes of each tribe with more than one member were aligned with MAFFT v7.310 (Katoh and Standley 2013) to create a multiple sequence that was then used as input to hmmbuild from HMMER v3.2.1 (Wheeler and Eddy 2013) to create a an HMM model per tribe. Each of these models were used to search throughout the predicted secreted proteins with a e-value of 0.01 for RxLR in the new genomes. In addition, HHBlits v3.1.0 (Remmert *et al.* 2011) was used in combination with the clustered and deeply annotated Uniclust30 database v2018_08 (Mirdita *et al.* 2017). HHblits is the HMM-HMM-based iterative sequence search that was shown to be very sensitive in detecting three-dimensional homologous proteins. Thus, the predicted secreted proteins were evaluated with HHBlits for structurally similar RxLR effectors in the new genomes compared to well annotated and validated RxLR effectors. Hits with the previous *P. megakarya* or *P. palmivora* proteins or with RxLR-like proteins were discarded. To predict the Crinklers effectors we took previously annotated and published genes Crinklers (Haas *et al.* 2009; Lamour *et al.* 2012; Ali *et al.* 2017), grouped into tribes, created HMM models per tribe and predicted the effectors based on the models in the same way than described above for the RxLRs.

### Identification of orthologs and duplicates

The software Orthofinder v2.2.6 (Emms and Kelly 2019) was used to predict orthologs across the seven *Phythophtora* species. Orthofinder was used with BLASTP for the sequence search, MAFFT v7.310 (Katoh and Standley 2013) for the multiple alignments, FastTree v2.1.10 (Price *et al.* 2010) for the tree inference method and an inflation parameter of 1.5. Within-species duplicates were defined as genes with multiple genes per paralogous groups were combined genes of the same species that had 50% or higher sequence identity, and both query and subject length coverage higher than 50% using BLASTP for the alignments. Once the duplicates were defined, a pair-wise homologous list was created of all the duplicates per species. This list was used as input for MCScanX (Wang *et al.* 2012) to classify the duplicates into dispersed, tandem, blocks or tandem-blocks.

### Synonymous substitution rates across duplicates

Proteins with a within-species duplicate of 50% identity or higher were used to calculate synonymous mutations. Each duplicate sequence was aligned using MAFFT v7.310 (Katoh and Standley 2013) and the alignments were edited with gBlocks v0.91b (Castresana 2000), to remove any gaps and keep the conserved blocks. The results format gBlocks edited and used as input into KaKs_Calculator v2.0 (Zhang *et al.* 2006). The *γ*-MYN algorithm was used as the method to estimate Ks using the standart genetic code table.

### Phylogenetic analyses

Multiple sequences were aligned with MAFFT v7.310 (Katoh and Standley 2013) and edited by gBlocks v0.91b (Castresana 2000). The clock-calibrated phylogenetic done as in (Matari and Blair 2014) using BEAST v1.10.4 (Drummond and Rambaut 2015). Briefly, the WAG substitution model was used, the Yule speciation process was assumed with a uniform distribution on the birthrate (0–100; initial value 0.01), while a strict clock was modeled with an exponential prior distribution (mean 1.0, initial value 0.01). The mean and 95% CI divergence time values of the strict clock estimation reported in (Matari and Blair 2014) were used as priors to calibrate our phylogenetic trees. A total of 10 million generations were created by tree. The clock calibrated tree in combination with the number of genes per gene family were used to study the evolution genes families across multiple *Phytophthora* species with the software CAFE v4.2.1 (https://github.com/hahnlab/CAFE) (De Bie *et al.* 2006). CAFE was run with default parameters optimizing the lambda parameter (option -s) to 0.033987 with a *P*-value cutoff of 0.01 (option -p).

### SNPs prediction and analyses

Paired-end reads of 150 bp were trimmed with Trimmomatic v0.36 (Bolger *et al.* 2014) and mapped to the reference genome using BWA mem v0.7.12-r1039 (Li and Durbin 2010). Duplicated mapped reads reads were removed, the remaining were tagged and used to predict SNPs with GATK’s UnifiedGenotyper (McKenna *et al.* 2010) with a minimum base score of 20 and ploidy of 2. The positions with a lower than 5 reads supporting it or higher than 1.5x the median coverage were removed for the downstream analysis. The predicted SNPs were used as input to GATKs’ “FastaAlternateReferenceMaker” v3.5-0-g36282e4 (McKenna *et al.* 2010) to generate alternative genomes per isolate and genes were extracted using the reference coordinates. Each gene sequence per isolate was aligned with ClustalW v2.1 (Thompson *et al.* 2003). The multiple alignment was as input to YN00 from the PAML v4.9f package (Yang 2007) with the universal genetic code to caculate the Omega values per genes. The VCF files with all sites and with all isolates per species were also used to calculate the Tajimas’D using 500 bp windows and sliding 250 bp with VCF-KIT v0.1.6 (Cook and Andersen 2017). The VCF files were also used in SnpEff v4.3t 2017-11-24 (Cingolani *et al.* 2012) to predict the genes with a gained stop codon.

### Gene expression analysis

The same filtered paired-end RNAseq reads used in the gene prediction were used for the gene expression analysis. The mycelia and zoospore reads were mapped to each species genome with the splice-aware mapper HISAT2 v2.0.5 (Kim *et al.* 2019) using the pair-end mode and with the following arguments: “-k 1–non-deterministic”. The *in planta* samples (*i.e.*, 15 hpi and 36 hpi) were mapped to a database that included the corresponding *Phytophthora* species in combination with the *T. cacao* criollo v2 genome (Argout *et al.* 2017). The count of the reads was done using the gff3 files with the “summarizeOverlaps” from the R package GenomicAlignments v1.18.1 (Lawrence *et al.* 2013), to extract the reads in the predicted exons. Estimation and statistical analysis of expression level using the count data of each gene with four replicates for each library were performed using the DESeq2 package in the R statistics suite (Anders and Huber 2010). For DESeq2’s default normalization method, scaling factors are calculated for each lane as median of the ratio, for each gene, of its read count of its geometric mean across all lanes and apply to all read counts. The raw and normalized counts of the gene expression values can be found in File S3.

### Data availability

The long-read, short-read and RNAseq data generated in this study have been submitted to the NCBI BioProject database under accession number PRJNA578180 (https://www.ncbi.nlm.nih.gov/bioproject/PRJNA578180). A genome browser for both species, with annotations, and an associated blast tool are available at http://www.cacaopathogenomics.com. Supplemental material available at figshare: https://doi.org/10.25387/g3.11983986.

## Results

### Phased assembly of long reads reveals the unique genome architecture of P. megakarya

Single molecule real-time long-read sequences were used to produce phased genome assemblies for three *P. megakarya* and three *P. palmivora* isolates collected from cacao plantations in Western Africa (Pm1, Pm4, Pm15, Pp2; Pm = *P. megakarya*; Pp = *P. palmivora*), Central America (Pp3), and Southeast Asia (Pp15) (Table 1, Tables S1, S3 and S4). Gene models were predicted using as transcriptional evidence RNA-seq data obtained from zoospores, mycelia, and infected plant material. All genome assemblies had a median coverage higher than 150x, high sequence accuracy (>99.999%), and gene space completeness comparable with other *Phytophthora* species assemblies (Table S5). A genome browser for both species, with annotations, and an associated blast tool are available at http://www.cacaopathogenomics.com.

**Table 1:** Genome assembly statistics for *P. megakarya* and *P. palmivora*. Average values ± SD are shown

Metrics	*P. megakarya*	*P. palmivora*
Size (MB)	222.04 ± 25.19	135.32 ± 17.21
Number of contigs	478.67 ± 155.32	169.67 ± 61.08
N50 length (Mb)	1.02 ± 0.56	1.53 ± 0.56
GC percentage	0.49 ± 0.01	0.49 ± 0

The average sizes of the highly contiguous assemblies were 222.0 ± 25.2 Mbp and 135.3 ± 17.2 Mbp for *P. megakarya* and *P. palmivora*, respectively (Figure 1D and Table 1). Genome sizes of both species were confirmed using multiple assembly methods and flow cytometry (Figure 1E and F; Table S6 and S7). The differences between the haploid genome assembly sizes and the inferred 1C-values estimated by flow cytometry were less than 15% (*P. megakarya*: 14.7 ± 5.6 Mb, *P. palmivora*: 13.5 ± 3.7 Mb). The similar values of the haploid genome assembly with the 1C-values validated the assembly sizes and suggests that both species are diploid (Ali *et al.* 2017). Flow cytometry confirmed the genome size in additional isolates of both species (Figure 1F; Table S6).

We then compared the *P. palmivora* and *P. megakarya* genomes with previously published genomes of *Phytophthora* species (Table S2). Although *P. megakarya* and *P. infestans* have similarly sized genomes, the structure of *P. megakarya* was strikingly different from *P. infestans* and any other *Phytophthora* genome. *P. infestans*’s large genome size (229 Mb) was caused by the proliferation of TEs, which account for up to 70% of its genome (Figure 1D; (Haas *et al.* 2009), which led to frequent gene-sparse regions with a large amount of intergenic space (Figure 1G). Despite a larger genome, the amount of repetitive content in *P. megakarya* (36.8 ± 1.2%) is similar to *P. palmivora* (33.5 ± 1.6%) and other *Phytophthora* spp. (25.5 ± 6.2%; Figure 1G, Table S8).

Interestingly, the genomes of both cacao black pod pathogens have more protein-coding genes than other *Phytophthora* species. The primary assemblies of *P. megakarya* and *P. palmivora* were predicted to have 57,577 ± 7,904 and 36,778 ± 4,481 protein-coding genes, respectively (Figure 1I, File S1). A high rate of duplication for eukaryotic universal single-copy orthologs (BUSCO) genes was observed; 36.7 ± 8.1% and 54.0 ± 11.3% of BUSCO genes were duplicated in *P. megakarya* and *P. palmivora*, respectively. On average, only 11% of BUSCO genes were duplicated in other *Phytophthora* genomes analyzed using the same methods (Table S5). The large percentage of duplicated conserved genes suggests that large-scale duplication processes occurred in the *P. megakarya* and *P. palmivora* genomes. Similar to the smaller genomes of other *Phytophthora* spp., and in contrast to the gene-dispersed genome of *P. infestans*, *P. megakarya* has a high gene density despite its larger size (Figure 1J). Overall, *P. megakarya* has a highly dense genome with high frequency of genes in even the more dispersed sections of its genome and *P. palmivora* has a relatively compact genome, with most genes within 1 kbp of each other. Unlike *P. infestans*, neither *P. megakarya* nor *P. palmivora* contain highly expanded regions with intergenic spaces greater than 20 kbp.

### Recent independent whole-genome duplications in P. megakarya and P. palmivora

**Figure 2:**
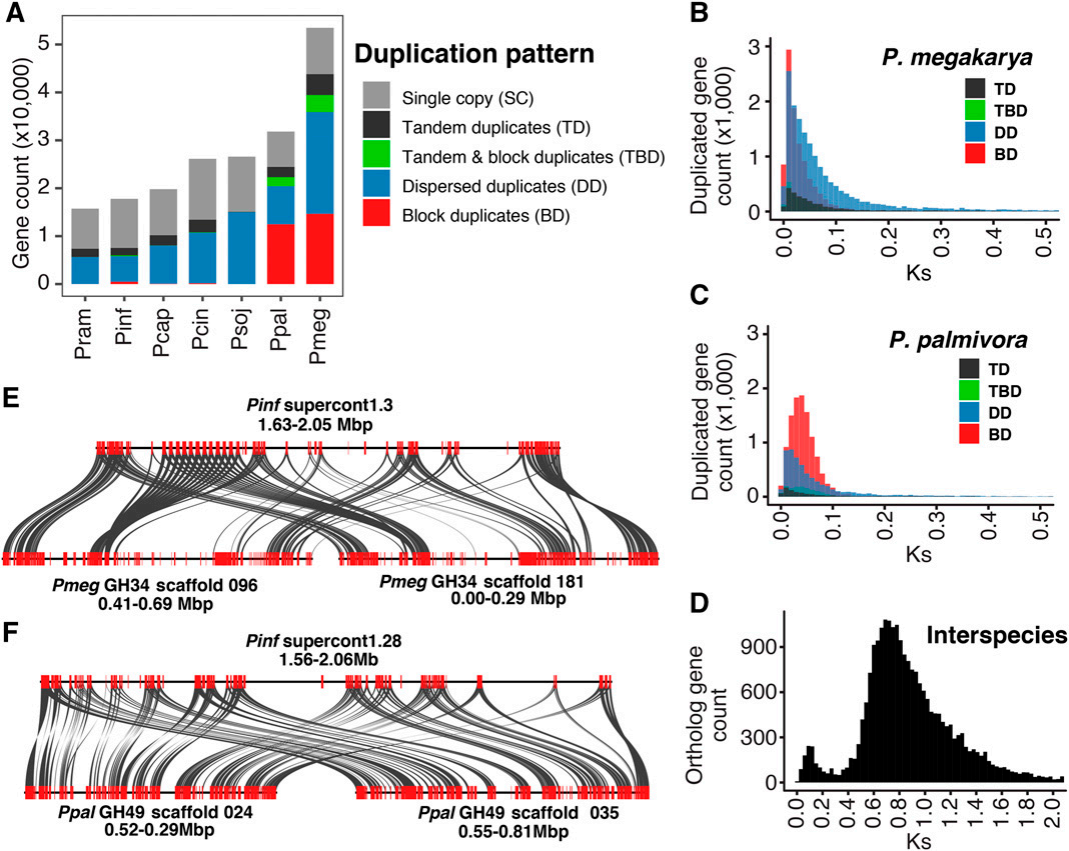
(A) Gene count per species divided by detected duplication patterns or the single copy category. Plots showing the synonymous mutations (Ks) of paralogs in *P. megakarya* (B) and *P. palmivora* (C). Synteny of duplicated blocks in *P. megakarya* (E) and in *P. palmivora* (F) with only one corresponding block in *P. infestans*. (D) Plot showing orthologs Ks distribution between *P. megakarya* and *P. palmivora*.

The large number of genes in *P. megakarya* and *P. palmivora* and the high rate of BUSCO gene duplication suggest a large-scale duplication process. Analysis of gene duplication across the entire gene space revealed 48,850 ± 9,475 (84.4 ± 4.5%) and 31,263 ± 6,015 (84.5 ± 6.6%) duplicated protein-coding genes in *P. megakarya* and *P. palmivora*, respectively, compared to 10,744 ± 3,461 (49.87 ± 5.39%) duplicated genes in other *Phytophthora* genomes (Figure 2A). We further classified duplications into the following patterns: conserved block duplicates (BD, minimum 5 genes), dispersed duplicates (DD), tandem duplicates (TD), genes in tandem and in block duplicates (BTD) or single-copy genes (SC). In both species, over 12,000 duplicated genes were organized in colinear BD genes (Figure 2A), with each block including a median of 8 genes, but the two species greatly differed in the number of DD genes (Figure 2A). Over 20,000 DD genes, about 40% of its genes, were found in *P. megakarya*. In contrast, ∼8,000 DD genes, only 25% of its genes, were found in *P. palmivora*. In both *P. palmivora* and *P. megakarya*, duplicated blocks were often on the same scaffolds and 100 kbp long, on average. Importantly, the presence of colinear duplicated blocks on the same scaffolds found both in the primary assembly and the haplotigs, confirmed the diploidy of the two species and ruled out colinear blocks belonging to homologous chromosomes (Figure S1). BD genes were virtually absent in the other *Phytophthora* genomes studied, with a maximum of only 476 BD genes in *P. infestans*.

The large number of colinear BD genes suggests that a recent whole-genome duplication (WGD) happened in the two cacao pathogens. The inference of WGD in *P. megakarya* and *P. palmivora* was supported by the synonymous substitutions rate (Ks) distribution across paralogous genes (Figure 2B&C). Well-defined peaks in the Ks distributions were evident in both species indicating a sudden increase in new genes for *P. megakarya* (0.021 Ks) and for *P. palmivora* (0.040 Ks) (Figure 2B&C). The large-scale duplication events appear to have happened independently and after speciation (0.820 Ks; Figure 2D). To estimate when the WGDs occurred, we searched for single-copy genes in other *Phytophthora* species that were present as two copies in *P. megakarya* and *P. palmivora*. Of these, only the genes in conserved colinear blocks were considered further, since they were likely generated during a WGD event. By this standard, a WGD event was estimated to occur 1.53 million years ago (MYA) in *P. palmivora* and 0.88 MYA in *P. megakarya* (Figure 1C, Figure S2). Since the divergence of *P. megakarya* and *P. palmivora* was estimated ∼16.4 MYA (Figure 1C), we conclude that the WGD events happened independently and after the two species diverged.

Nearly 60% of all the genes were duplicated as part of the large-scale duplication process (<0.5 Ks) in both species, representing approximately 75% of the gene families in both species (Figure S2 and File S1). In addition, several complete duplicated blocks in *P. megakarya* and *P. palmivora* were colinear with a single block in *P. infestans* (Figure 2E&F; Figure S3). These results are strong evidence that independent WGD events occurred in both *P. palmivora* and *P. megakarya*.

### Extensive gene expansion and duplicate dispersion in P. megakarya

Our analyses show that both *P. megakarya* and *P. palmivora* have experienced recent WGD events, but these events do not explain their 40% difference in annotated protein coding genes. To better understand this difference, we focused on the number and organization of gene families across multiple *Phytophthora* species (Table S2). Using published gene models for comparison, we found nearly 3,000 more gene families expanded in *P. megakarya* and *P. palmivora* than the median number of families expanded in other *Phytophthora* species (*e.g.*, *P. infestans*: 960; Figure 1C). Though the number of expanded gene families was similar for the two cacao pathogens, the number of predicted genes gained was much larger in *P. megakarya* (27,166) than in *P. palmivora* (9,546).

Considering the large number of gene families expanded in these two species, hereinafter we focused on the gene families with at least a twofold increase when compared to the median of the other *Phytophthora* species and containing at least 10 genes. The majority of these families were in *P. megakarya* with 168 families (including 9,210 genes), while *P. palmivora* only had 96 such families (including 3,404 genes). The difference in the number of genes in the > twofold expanded families between *P. megakarya* and *P. palmivora* indicates that there was a unique expansion of relatively few gene families in *P. megakarya*.

We then studied the contribution of the duplication patterns to the > twofold expanded families in *P. megakarya* and *P. palmivora*. The BD, TD, TBD and SC categories were relatively similar in both species, only differing by a maximum of 898 genes. However, *P. megakarya* had 3,341 more DD genes than *P. palmivora*, suggesting that DD genes contributed disproportionately to the expansion of genes families in *P. megakarya* and to its total number of genes. The mechanism by which duplication may have occurred was examined next.

### Expanded gene families are generally associated with transposable elements

**Figure 3:**
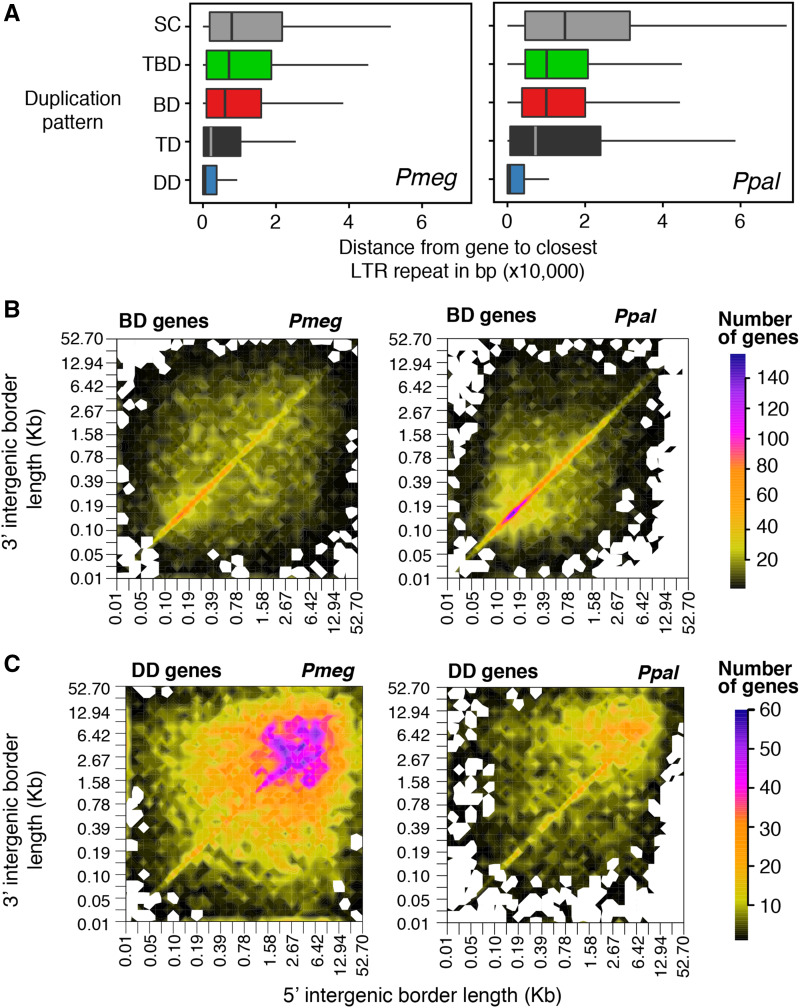
(A) Distribution of the distance between the duplication patterns and LTR TEs. (B) Intergenic space heatmap of genes in block duplicated genes (C) and dispersed duplicated genes. *Pmeg: P. megakarya* and* Ppal: P. palmivora*.

TEs have the ability to mediate gene duplications of the host genes and the formation of the new genes (Volff 2006; Xiao *et al.* 2008; Tan *et al.* 2016). The proliferation of Long Terminal Repeat (LTR) TEs was reported to be the main cause of the genome expansion in *P. infestans*, contributing to regions in the genome where gene order was not conserved, and the expansion of intergenic space (Haas *et al.* 2009). LTRs account for the large majority of the repetitive elements in the genome in *P. megakarya* (76.3%) and *P. palmivora* (75.0%) (File S2). Consequently, we studied the relationship between duplication patterns and LTRs by calculating the distance between genes and these elements (Figure 3A). We found that DD genes were significantly closer (Kolmogorov-Smirnov test, *P-value <* 2.2e-16) to LTRs than to any other duplication category of genes in both species.

On average, we found 7.4 LTR elements within 10 kbp (up or downstream) from the DD genes compared to only 4.5 elements at that distance from the BD genes (Table S9). The LTRs were larger, on average, around DD (991.7 bp) and TD (949.1 bp) genes than when they were near other gene duplication categories (833.0 bp). As expected, the differential accumulation of repeats across the duplication patterns had an important impact on the intergenic space. The highest intergenic frequency of the BD genes was around 200 bp in both species (Figure 3B), while the intergenic space of the DD genes had the highest frequency around 4,500 bp. DD genes had an overall trend toward the most expanded intergenic regions of the genome in both species, but it was much more striking in *P. megakarya* (Figure 3C).

We then inspected the association between TEs and gene families unique to or dominated by *P. megakarya* and described their duplication patterns (File S2). We found gene family duplicates in repetitive regions that were very close to one another. For example, 69.4% of FA00226 family members were on scaffold 20 and all 53 members of family FA00514 were on scaffold 440. There were also instances of multiple highly expanded gene families, mainly non-expressed (*i.e.*, not detectable in our RNA-seq dataset; File S3), and hypothetical protein families, loosely grouping together throughout the dispersed regions of the genome. These families were frequently associated with and overlapped LTR/Gypsy TEs (*e.g.*: 1,120 members of FA00003 and 877 members of FA00005), but many other consistent associations were observed as well. This included associations between FA00460 and DNA/PiggyBac and between FA00326, FA00947, and Helitrons.

We observed another gene duplication pattern, so far unique to *P. megakarya*, which included multiple unique expanded gene families arranged in duplicated inverted blocks (*e.g.*: FA00074, FA00107, FA00211, and FA00254 and others; File S2). These families primarily consist of hypothetical proteins and are associated with DNA/PiggyBac-like TEs, and often include inverted repeats on the gene block ends and repeat sequences in-between (Figure S4). DNA/PiggyBac TEs create double stranded DNA breaks, leaving TTAA overhangs not requiring DNA synthesis for repair (Mitra *et al.* 2008). Related elements are found in diverse species (Sarkar *et al.* 2003), Inverted repeats are expected to cause instability and are possibly associated with the required chromosomal rearrangements needed to stabilize the genome after WGD.

Overall, when looking at many highly expanded gene families in *P. megakarya* and *P. palmivora*, we observed a high number of hypothetical protein-coding genes, their often-close association with TEs (File S2), and a tendency to be non-detectable in the RNA-seq data we collected, which suggests low or no expression (File S3). Duplications of functional gene families have long been loosely associated with TE-driven genome expansion (Jiang *et al.* 2006; Jiang and Tyler 2012), potentially carried along in part or entirely to new location in the genome, and subject to potential modification in the process. This would appear to be a major difference among the expanded gene families.

### Genome duplication and expansion lead to exceptional effector content in P. megakarya

**Figure 4:**
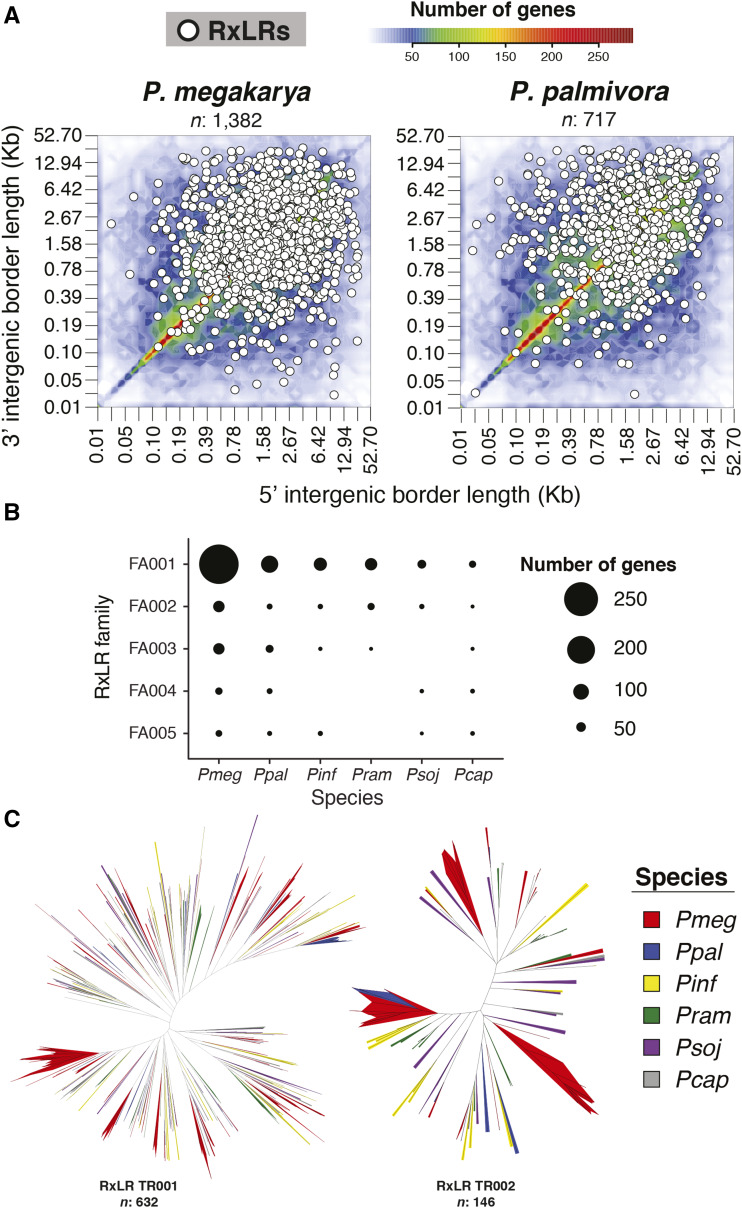
(A) Intergenic space heatmap of *P. megakarya* and *P. palmivora* with an overlayed relative position of the predicted RxLR effectors. (B) Numbers of genes in the top five RxLR families per species. (C) Phylogenetic analysis created by resampling the site likelihoods 1,000 times of the two largest families across multiple species of *Phytophthora* showing a clear branch-specific amplification pattern in *P. megakarya*.* Pinf: P. infestans, Pmeg: P. megakarya, Ppal: P. palmivora, Psoj: P. sojae, Pram: P. ramorum* and* Pcap: P. capsici.*

Overall, both species were predicted to have exceptionally large number of RxLR effectors; a total of 1,382 were predicted in *P. megakarya*, 717 in *P. palmivora* (Figure 4A). Although the largest RxLR families were expanded in both pathogens, this was clearer in *P. megakarya* than in *P. palmivora* (Figure 4B). In addition, there were many “putative effectors” sharing homology with and often bordering RxLRs in both species (561 in *P. megakarya* and 251 in *P. palmivora*) (File S4). These putative effectors, though incomplete, are candidates for evolving or degenerating RxLRs, as is to be expected considering the expanded RxLR numbers in these two species.

A phylogenetic analysis of the two largest RxLR families in multiple *Phytophthora* species showed a pattern of branch-specific duplications within each gene family and the most dramatic expansions in *P. megakarya* (Figure 4C). The most expanded RxLR gene family (RxLR-FA001) accounted for 21.4% and 15.6% of the RxLRs in *P. megakarya* and *P. palmivora*, respectively. Many of the RxLR effectors shown in Figure 4C were found in the expanded gene-sparse regions of the genome in both pathogens. However, in contrast to the pattern of intergenic space surrounding dispersed duplicated genes in general (Figure 3C), many RxLR effectors were also abundant in the more compact (intergenic space ≤ 10 kbp) and gene-rich regions of the genome (Figure 4A). This suggests that both species have evolved large effector repertoires by both WGD and expansion.

Other gene families with virulence related functions were also detected among the expanded families. Necrosis inducing proteins (NPP1, FA00029) and a family of bZIP transcription factors (FA00017) required for zoospore motility and plant infection (Blanco and Judelson 2005; Gamboa-Meléndez *et al.* 2013), are known to be expanded in *P. sojae* (Ye *et al.* 2013), and were also within the top 10 most-expanded gene families in both species. In *P. megakarya*, a serine/threonine protein kinases family (FA00009) expanded to a size 169 genes larger than other species. These kinases are important for zoospore release, zoospore viability, encystment, and cyst germination and could be related to the higher sporulation ability of *P. megakarya* (Judelson and Roberts 2002).

Genes within RxLR families that were unique to or expanded in *P. megakarya* (FA047, FA107, FA147) or *P. palmivora* (FA033, FA153, FA179) were induced *in planta* or in zoospores based on RNA-seq transcriptome profiling. At least 1,058 of 1,381 *P. megakarya* Pm1 RxLRs and 540 of 717 *P. palmivora* Pp2 RxLRs were transcriptionally active (Table S10, Figures S5-S7, File S3). Twenty-two RxLRs in *P. megakarya* and 32 RxLRs in *P. palmivora* were consistently detected as up-regulated *in planta* compared to their expression in mycelia or zoospores (Figure S8).

Together, these data show that genome duplication and gene family expansion led to increase of genes with functions related to virulence, potentially enabling these pathogens to adapt to novel circumstances.

### Genetic signatures point to adaptive evolution in the expanded gene families

Genetic analyses of multiple individuals are necessary to evaluate the evolutionary effects of WGD and gene family expansion in *P. palmivora* and *P. megakarya*. Thus, we resequenced fifteen isolates of *P. megakarya* from three West African countries and 18 isolates of *P. palmivora* (Table S1 and Figure S9). Synonymous (*d*_S_) and nonsynonymous (*d*_N_) substitutions rates were estimated in pairwise comparisons of individuals to get an Omega ratio (ω *= d*_N_*/d*_S_). Genes with an ω > 1 have been associated with positive selection (Yang and Bielawski 2000; Kelley and Swanson 2008). In total, 11,716 *P. megakarya* Pm1 genes and 6,567 *P. palmivora* Pp2 genes had ω > 1 and potentially under positive selection. In both species, single-copy genes conserved in the seven *Phytophthora* species, which are expected to be under neutral selection, showed ω values significantly lower than 1 (One sample *t*-test, *P* < 2.2 × 10^−16^).

We then tested for evolutionary signal enrichment among > twofold expanded families with the Bonferroni-corrected Fisher’s Exact Test (BCFET). Remarkably, genes under positive selection in both cacao pathogens were significantly enriched in the largest RxLR gene family (“FA00008”, BCFET *P* < 2.01 × 10^−10^), an expanded bZIP transcription factor family (“FA00017”, BCFET *P* < 1.11 × 10^−9^), and an expanded M96 mating-specific family (“FA00043”, BCFET *P* < 1.30 × 10^−2^). The significant enrichments of positively selected genes in specific highly expanded families strongly suggests that their expansion is evolutionarily beneficial.

SNPs in the population associated with premature stop codons were screened to identify genes likely under relaxed selection. We found 10,702 and 5,289 genes with a gained stop codon in *P. megakarya* Pm1 and *P. palmivora* Pp2, respectively. The RxLR-encoding family (“FA0008”) was enriched in genes potentially under relaxed selection in both species (BCFET *P* < 1.73 × 10^-2^). Among the families enriched in genes potentially under relaxed selection, we also found an additional expanded RxLR family in *P. megakarya* (“FA00137”, BCFET *P* < 1.30 × 10^−2^) and an expanded CRN effector family in *P. palmivora* (“FA00255”, BCFET *P* < 3.34 × 10^−2^). However, most (∼80%) of the families significantly enriched with premature gained stopped codons (BCFET *P* < 0.05) and potentially under relaxed selection were designated “hypothetical protein”. The large number of genes in expanded families with predicted premature gained stop codons suggests that relaxed selection is permitted by large-scale duplicative processes that increase redundancy.

## Discussion

The genomes of *P. megakarya* and *P. palmivora* revealed information about their evolution and significant differences between the two pathogens that might explain the high virulence of *P. megakarya* in cacao compared to *P. palmivora*. Our analyses support the hypothesis that *P. megakarya*, like *P. palmivora* (4), also underwent WGD. Evidence of WGD includes (*i*) genomes larger than other *Phytophthora* species, which was confirmed for multiple isolates and validated with flow cytometry, (*ii*) a high number of genes duplicated in conserved colinear blocks, (*iii*) a large number of expanded gene families, and (*iv*) the distribution of Ks mutations around a single event. Together, the flow cytometry results and genome structure confirmed that both species are diploid and that WGD was followed by diploidization for both species (*i.e.*: paleopolyploids)(Wolfe 2001). The differences in the number of chromosomes between *P. megakarya* (5-6 chromosomes) and *P. palmivora* (9-12 chromosomes) may have to do with the re-diploidization process after the WGD event (Sansome *et al.* 1975; Brasier and Griffin 1979).

A WGD event can occur as autopolyploidy by doubling the copy number of each chromosome, or as allopolyploidy by the hybridization of two different species (Kihara and Ono 1926; Wolfe 2001). Both allo- and autopolyploidization have been reported in some *Phytophthora* species (Sansome 1977; Bertier *et al.* 2013). Nonetheless, inter-species hybridization of *P. megakarya* or *P. palmivora* have not been observed. The single peak in the Ks distributions in both species does not suggest that genome duplication was caused by the hybridization of distinct species, but rather that WGD was due to autopolyploidization. Our estimates suggest that the WGD event happened independently and much later than the divergence between the species, with the *P. palmivora* duplication occurring ∼0.65 MYA before *P. megakarya*. The timing of the WGD events provides an opportunity to study how recent WGD may influence pathogen virulence. Polyploid isolates of *P. infestans* (Sansome 1977) and hybrid *P. alni* subsp. *alni* (Redondo *et al.* 2015) are reportedly better adapted to specific environments than diploid *Phytophthora*. In plants, the incidence of polyploidy is greater in populations subjected to environmental challenges like high altitudes and large-scale climatic perturbation (Ramsey and Schemske 1998). The ability of the polyploids to occupy new habitats is well-documented in plants and fishes (De Bodt *et al.* 2005; Santini *et al.* 2009; Ramsey 2011); while it has been associated with the expansion of host range in plant pathogens (Depotter *et al.* 2016). WGD and the subsequent modification of copied sequences can enhance genetic diversity and be advantageous if accompanied by neo- and sub-functionalization (Sémon and Wolfe 2007; Martens *et al.* 2008; Buggs *et al.* 2011; Berthelot *et al.* 2014; Hughes *et al.* 2014). For example, in maize nearly 13% of duplicated genes after a WGD have functionally divergent regulatory regions (*i.e.*, neo-functionalization) and are expressed at novel times and environments (Hughes *et al.* 2014), while an ancient WGD in ray-finned fishes is reportedly responsible for over 10% of the biodiversity in this group of fishes (Santini *et al.* 2009).

In *P. megakarya* and *P. palmivora*, we show that much larger families of virulence factor genes (4) compared to other *Phytophthora* species (Fellbrich *et al.* 2002; Dou *et al.* 2008; Jiang *et al.* 2008; Haas *et al.* 2009), including RxLR, CRN and NPP1 effectors, are the result of WGD and gene expansion. Remarkably, the process of WGD and gene family expansion makes *P. megakarya* the *Phytophthora* species with the highest number of RxLRs effectors observed so far. Effectors facilitate host colonization by modulating the plant immunity (Toruño *et al.* 2016). Effector genes often undergo rapid changes in pathogen populations to overcome newly-evolved host resistance molecular mechanisms, thus the rapid effector diversification is a crucial component of pathogen success (Fouché *et al.* 2018; Sánchez-Vallet *et al.* 2018). Our population genetic analysis confirms that the largest families of RxLRs and other expanded families in both species were overrepresented among genes under positive selection. This suggests that these genes and the gene family expansion participate in the adaptive evolution of the pathogens by creating new beneficial traits in the population (Emerson and Thomas 2009; Smadja *et al.* 2009). Similar results have been observed in *P. sojae* and *P. ramorum*, in which the C-terminal effector domain of the RxLRs proteins are under positive selection (Win *et al.* 2007). Genes under positive selection can confer essential traits, like the ability to escape host recognition through the novel versions of the effector protein, as shown in the flax rust fungus (*Melampsora lini*) (Dodds *et al.* 2006). In the *Phytophthora* genus, potential disease-related genes like the phytotoxin-like scr74 gene family in *P. infestans* (Liu *et al.* 2005) and effectors from the CRN family in *P. sojae* (Shen *et al.* 2013) have also been reported to be under positive selection. Unlike *P. palmivora*, which has a global presence and broad host range, *P. megakarya* is confined to West Africa and causes economic disease losses only on cacao (Bailey *et al.* 2016). Despite this apparent specificity, *P. megakarya* can associate with and has been isolated from the roots of many native tree species (Akrofi 2015). Since cacao was introduced in West Africa less than 200 years ago, most of the adaptive events resulting in *P. megakarya*’s genomic structure likely occurred on native hosts prior to its encounter with cacao.

Although we found evidence for WGD duplication events for both species, we show that *P. megakarya* has a larger genome, a greater number of genes, and a greater number of duplicated genes that are found dispersed in the genome. Dispersed duplicated genes account for most of the difference between the number of genes and genome sizes of *P. megakarya* and *P. palmivora*. Most dispersed duplicates were tightly associated with transposable elements and tended to occur in genomic regions with the largest intergenic space. Genes with virulence-related functions were also often located in gene-sparse regions of the genome. The manner in which the dispersed duplicates are evolving is consistent with the two-speed genome mode of evolution described in other eukaryotic pathogens (Dong *et al.* 2015; Faino *et al.* 2016), in which the gene-sparse and repeat-rich regions of the genome are the largest sources of novel functions. However, despite the great number of dispersed duplicated genes and a likely contribution of TEs in their dispersion, the intergenic space of *P. megakarya* is significantly smaller than that of *P. infestans*. Thus, the genome of *P. megakarya* exhibits a unique architecture with a size of 222 Mbp and intergenic space smaller than 20 kbp. The unique combination of WGD and large-scale transposable element driven gene/genome expansion has given *P. megakarya* one of the largest genomes, number of predicted genes, and candidate effector pools among *Phytophthora* species known to date. The combination of a genome size twice that of *P. palmivora* and a chromosome number half that found in *P. palmivora* manifests itself in the extra-large chromosomes, which is key to *P. megakarya’s* identification and recognized in the species name “megakarya” so many years ago (Sansome *et al.* 1975; Brasier and Griffin 1979).

This study constitutes a significant step toward unraveling the virulence differences in consequential *Phytophthora* pathogens, *P. megakarya* and *P. palmivora*. The screening of isolates based on the effector repertoire discussed here can inform breeding programs and monitor pathogen evolution and epidemics. Additional research is required to determine the molecular causes of differences in virulence between these species and the basis of *P. megakarya* aggression. The resources produced by this study should be helpful in the pursuit of this goal.
